# Weight Status, Adherence to the Mediterranean Diet, and Physical Fitness in Spanish Children and Adolescents: The Active Health Study

**DOI:** 10.3390/nu12061680

**Published:** 2020-06-04

**Authors:** Samuel Manzano-Carrasco, Jose Luis Felipe, Javier Sanchez-Sanchez, Antonio Hernandez-Martin, Leonor Gallardo, Jorge Garcia-Unanue

**Affiliations:** 1 Faculty of Sport Sciences, University of Castilla-La Mancha, 45004 Toledo, Spain; Samuel.Manzano@uclm.es (S.M.-C.); Antonio.HMartinSan@uclm.es (A.H.-M.); Leonor.Gallardo@uclm.es (L.G.); Jorge.GarciaUnanue@uclm.es (J.G.-U.); 2 School of Sport Sciences, Universidad Europea de Madrid, 28670 Madrid, Spain; javier.sanchez2@universidadeuropea.es

**Keywords:** nutrition, health, physical activity, lifestyle, obesity, sedentary lifestyle

## Abstract

The aim of this study was to analyze the differences in body composition and physical fitness according to the weight status (normoweight, overweight and obese) and the level of adherence to the Mediterranean diet (MD; low, medium or high), in physically active children and adolescents. Furthermore, this study also analyzed the relationship between body composition and physical fitness with Body Mass Index (BMI), fat mass and the level of adherence to the MD. In total, 1676 participants aged 6–17 from different municipal sports schools participated in this cross-sectional study. Data on adherence to the MD (a KIDMED questionnaire), anthropometric measurements, body composition and physical fitness parameters (the 20-m shuttle run test and muscular strength) were collected. A total of 43.5% of the sample were presented as overweight and obese, and only 35.7% had high or optimal adherence to the MD. The results revealed that a normoweight status was associated with greater cardiorespiratory fitness (*p* < 0.05; ES: 0.50 to 0.67) and lower-body muscular strength (*p* < 0.05; ES: 0.58 to 1.10). The overweight group showed more significant results than the other groups in handgrip strength (*p* < 0.01). Greater adherence to the MD in this population indicated better physical fitness, but only in boys. It is concluded that normoweight status and optimal adherence to the MD in children and adolescents are associated with health benefits, which are significant in the body composition and the effect on physical fitness.

## 1. Introduction

The prevalence of overweightness and obesity in children and adolescents is a global public health problem [1,2]. In 2016, a total of 213 million people in this population were overweight, and the global rates of obesity in children and adolescents from 5 to 19 years of age have multiplied by 10 times worldwide. In 2022, there will be more children and adolescents with obesity than those who are underweight [3]. This epidemic represents a significant threat to public healthcare (i.e., associated healthcare costs) and, after several decades of fighting it, one of the greatest public health challenges of the twenty-first century remains the struggle against the increase in obesity in this population [4], with the additional problem of maintaining increasing rate trends in the future [5].

Currently, obesity is one of the most serious non-communicable diseases (NCDs), classified as a chronic disease of a multifactorial origin [3]. These NCDs are the result of a combination of different factors, physiological, environmental, genetic and, behavioral, showing to be long-lasting over time [6]. The clinical manifestations of NCDs generally appear during adulthood; however, they usually start to develop at an early age [7]. Thereby, childhood and adolescence are key stages in the acquisition and establishment of lifestyle habits and a significant number of physiological and psychological changes that will take place during adulthood [8]. In these periods of life, several factors influence health status, such as dietary patterns and the daily practice of physical activity. Both factors are fundamental to maintaining a healthy lifestyle, which allows us to acquire healthy eating habits and an adequate level of physical fitness that will be the determinants of present and future health [9,10]. Therefore, at these ages it is fundamental to adopt these habits since they can be essential to prevent NCDs and can be as a predictor of health and subsequent morbidity and mortality [11].

Public health organizations seek to promote and develop healthy habits (i.e., daily physical activity, active commuting, a balanced diet, etc.) among children and adolescents, as they are key aspects for the prevention of obesity [12]. However, more than 80% of adolescents do not comply with the 60 daily minutes of moderate-to-vigorous intensity physical activity practice as recommended by the World Health Organization (WHO), with sedentarism and physical inactivity being the two major determining factors of lifestyle and health in children and adolescents [13]. In this sense, physical activity and healthy dietary habits are essential for greater health-related quality of life and physical fitness. The main factors of physical fitness (cardiorespiratory fitness, flexibility and muscular fitness) along with body composition are shown to be directly associated with improving health [14]. Besides, physical fitness is generally recognized as a powerful marker of health-related outcomes in both childhood and adulthood and a key determinant of current and future health status [9]. Thereby, high levels of physical activity and physical fitness in youth populations are considered fundamental elements for the maintenance of well-being and health, as well as a decrease in the risk of developing NCDs [15]. Conversely, low levels of physical fitness and insufficient physical activity in this population are associated with NCD risk factors for obesity even at young ages [16] and can persist into adulthood [17]. Therefore, the control and assessment of physical fitness in all populations are essential to detect and determine different public health policies for this age group.

In terms of eating habits, a higher prevalence of obesity is associated with inadequate nutrition in children and adolescents [18]. There are some useful questionnaires for evaluating eating behaviors in the general population and in the youth population (i.e., the Three-Factor Eating Questionnaire, the Children’s Eating Habits Questionnaire, etc.) [19,20]. However, more recently, instruments have been designed to evaluate the adherence to specific healthy eating patterns in this population, such as the Mediterranean diet (MD) [21], that are considered an essential type of diet for preventing and reducing obesity at this age [22]. The KIDMED score (Mediterranean Diet Quality Index) developed by Serra-Majem et al. [23] is a nutritional index that evaluates adherence to the MD and the quality of diet in children and adolescents. This type of diet is traditionally the one with the highest percentage of adherence in Spain [24] and coexist with other lesser-known dietary patterns such as the Atlantic diet in north-western Spain [25]. The MD is based on the low consumption of meat products; a moderate intake of milk, cheese and yogurt; a moderate to high intake and high intake of olive oil and fish; and a high intake of vegetables, fruits, legumes and unrefined cereals [26]. Nevertheless, the principles of the MD and the nutritional recommendations are not complied or followed by adolescents living in North America, Europe, or Oceania [27]. In recent years, the Mediterranean region has undergone a transition towards a more westernized diet, where there is a higher consumption of processed foods and animal products and a lower intake of plant-based food than has been recommended among the various Mediterranean countries [28]. Previous studies [29,30,31,32] have shown that greater adherence to the MD reportedly reduces the risk of metabolic syndrome, cardiovascular diseases, type II diabetes mellitus, neoplastic disease and overall mortality, and increases life expectancy. Given the importance of a healthy and balanced diet, together with an adequate level of physical fitness to prevent obesity and improve the health-related quality of life in this population, studies that address this issue are necessary and solutions or strategies must be proposed.

Therefore, the aim of this study was to analyze the differences in body composition and physical fitness according to the weight status (normoweight, overweight and obese) and the level of adherence to the MD (low, medium or high) in physically active children and adolescents. Furthermore, this study also analyzed the relationship between body composition and physical fitness with Body Mass Index (BMI), fat mass and the level of adherence to the MD.

## 2. Materials and Methods

### 2.1. Design and Participants

A cross-sectional and quantitative study was conducted with athletes who practice any sport modality at least two days a week for one hour, aged 6–17 years old (11.11 ± 2.62 years; 44.48 ± 15.25 kg; 147.51 ± 15.85 cm), during the 2018–2019 academic year from different municipal sports schools in Castilla-La Mancha (a central region of Spain). All athletes in the selected sports academies were invited to the study. The participants and their parents or legal guardians were previously informed through a document about the purpose of the study and the nature of the tests that would be performed, and an informed consent must have been signed prior to the tests. 

The final study population was formed of 1676 children and adolescents and divided into four subsamples as a function of sex and pubertal status. For the pubertal status, given the importance of maturity in this population [33], a Marshall and Tanner test [34] was conducted (stage I was categorized as prepubertal and stage II and III as pubertal). The final four subsamples were formed by 774 prepubertal boys (46.18% of the study population), 432 pubertal boys (25.78% of the study population), 335 prepubertal girls (19.99% of the study population), and 135 pubertal girls (8.05% of the study population). The participants were evaluated individually once before a training session. The tests lasted between 60 and 90 min and were carried out in groups of 12 to 14 athletes.

This research was carried out in compliance with the standards of the Declaration of Helsinki (2013 revision, Brazil) and following the guidelines of the European Community for Good Clinical Practice (111/3976/88 July 1990), as well as the Spanish legal framework for clinical research on humans (Royal Decree 561/1993 in clinical trials). The project was approved by the Bioethics Committee for Clinical Research of the Virgen de la Salud Hospital in Toledo and by the supervisors of the University of Castilla-La Mancha (Ref.: 508/17042020).

### 2.2. Procedures and Variables

#### 2.2.1. Weight Status Classification

Each of the four subsamples was divided into three groups based on their weight status, calculated using the BMI tables of the WHO for the international growth standards for school-aged children and adolescents [35]. The BMI was calculated with the weight (kg) divided by the squared height (in meters). Normoweight, overweight and obese were defined on the basis of a sex-specific z-score relative to the WHO BMI-for-age reference (i.e., BMI-for-age z-scores > + 1SD is classified as overweight, equivalent to BMI 25 kg/m^2^ at 19 years; and > + 2SD as obese, equivalent to BMI 30 kg/m^2^ at 19 years) [35].

#### 2.2.2. Level of Adherence to the Mediterranean Diet Classification

Each of the four sub-samples were also divided into three groups based on the level of adherence to the MD, classified by KIDMED questionnaire. This instrument has been created and validated for the same population by Serra-Majem et al. in the enKid study [23,36] in which a quantitative 169-item food-frequency questionnaire was administered to obtain a database to compare and validate KIDMED scores. More recently, this instrument has also been validated by the comparison with objective measures [37,38]. 

This instrument was designed specifically for youth and adolescent populations and is the most widely used tool to assess adherence to the MD according to various systematic reviews [27,39], which allows for obtaining a highly developed contextual framework to interpret the results. KIDMED consists of 16 items: 12 items represent a positive score for the adherence to the MD, and the remaining 4 items represent a negative score [23]. A positive answer to a question that involves greater adherence to the diet is worth +1 point (Q1–Q5, Q7–Q11, Q13 and Q15). A positive answer to a question that means less adherence to the diet is worth −1 point (Q6, Q12, Q14 and Q16). Negative answers do not score (a value of 0 is noted). The KIDMED score is the sum of all the scores and ranges from −4 to 12 points (minimum to maximum adherence to the MD). Furthermore, in addition to the KIDMED score, the main objective of this instrument is to classify the participants into three levels of adherence to the MD: low (≤ 3 points), medium or moderate (4–7 points), and high or optimal (≥8 points) [23].

#### 2.2.3. Body Composition Variables

Each participant performed an anthropometric evaluation at a 5-min interval with a protocol according to previous investigations [40]. A portable segmental analyzer of multifrequency body composition (Tanita MC-780, Tanita Corp., Tokyo, Japan) was used to measure weight (kg), total fat mass (kg and %), total lean mass (kg and %), and trunk fat mass (kg and %). Height (cm) was measured with a height rod (Seca 214, Hamburg, Germany). 

#### 2.2.4. Physical Fitness

The different parameters of physical fitness were assessed using a modified version of the extended Assessing Levels of Physical Activity (ALPHA) health-related fitness battery for children and adolescents [41]. The components are described as follows.

##### Cardiorespiratory Fitness

Cardiorespiratory fitness was measured using the Course–Navette test (20-m shuttle run test [20 mSRT]). In this test, the participants run in a straight line between two lines distanced 20 m apart, while keeping pace with the acoustic signals from a speakerphone audio player [42]. The initial speed is 8.5 km h^−1^, which is increased by 0.5 km h^−1^ each min [43]. The test is finished when the participant does not reach the line before the audio signal on two occasions or when the participant must stop because of fatigue. Therefore, the results were recorded in stages of 1 min duration. This test was performed last so that fatigue did not interfere with the normal development of the other tests, and the participants performed the test once. The results of the test were also standardized using the values of percentiles (pc) according to age and sex [44,45].

##### Muscular Fitness

Handgrip strength with hand dynamometer with adjustable grip (TKK 5001 Grip A; Tokyo, Japan) was used to evaluate upper-body muscular strength. Participants had to close their hands with maximum force continuously for 2 s with the elbow position in full extension. The test was repeated twice (right hand and left hand alternately). The best score from the dominant hand for each participant was taken to the nearest 1 g and recorded as kg [41]. On the other hand, a countermovement jumps test (CMJ) with arm swing was performed to evaluate lower-body muscular strength [46]. The height was calculated to the nearest 0.1 cm by photoelectric cells consisting of two parallel bars (Optojump, Microgate, Bolzano, Italy) and recorded as cm, which measure flight time taken as the duration between take-off and landing. The participants completed three attempts with 1 min of recovery and were instructed to jump as high as possible with a rapid, preparatory downward eccentric action, while the arms were freely able to be moved. The results of the handgrip strength and CMJ were also standardized using the values of percentiles (pc) according to age and sex [44,45].

### 2.3. Statistical Analysis

Data were presented as means ± standard deviations. A Kolmogorov–Smirnov distribution test was performed to confirm a normal distribution of the variables. Firstly, the four subsamples (prepubertal boys, pubertal boys, prepubertal girls and pubertal girls) were classified into three groups based on weight status (normoweight, overweight, and obese) and three groups based on the level of adherence to the MD (low, medium or high). A one-way ANOVA was used to evaluate the differences between the three groups of each classification. The post-hoc analysis was adjusted by the Bonferroni method. The test was replicated individually in the four subsamples. All body composition variables and physical fitness parameters were used as dependent variables. Furthermore, effect sizes (Cohen’s d, ES) were calculated and defined as follows: trivial, < 0.19; small, 0.2–0.49; medium, 0.5–0.79; large, > 0.8. All data were statistically analyzed using SPSS V24.0 for Windows (SPSS Inc., Chicago, IL, USA). Finally, several regression estimations were performed to analyze the relationship between body composition variables, physical fitness parameters and KIDMED score (−4 to 12) with BMI (kg/m^2^) and fat mass (%), and the relationship between body composition and physical fitness with KIDMED score (−4 to 12). To avoid multicollinearity problems, only the main body composition and physical fitness variables were used: 20 mSRT (stages), handgrip (kg) and CMJ (cm). All models were also controlled by sex, pubertal status and age. The regression did not present normality problems. Moreover, the Variance Inflation Factor (VIF) did not report any multicollinearity problems. However, a robust standard error must be used because of the presence of heteroscedasticity. Coefficients were reported as non-standardized and as beta standardized. The level of significance was set at *p* < 0.05.

## 3. Results

### 3.1. Differences in Body Composition and Physical Fitness between Weight Status Groups

**Figure 1:**
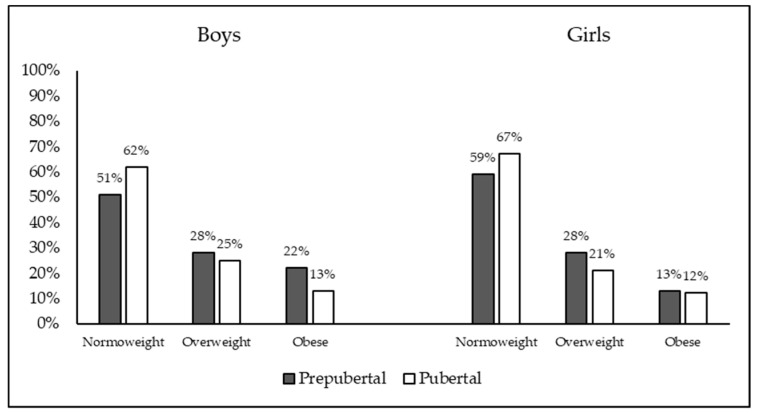
Percentage of the sample with different weight classifications as a function of sex and pubertal status.

After the classification based on the WHO BMI-for-age reference, 56.6% of the sample (*n* = 947) were classified as normoweight, 26.6% (*n* = 445) as overweight, and 16.9% (*n* = 284) as obese. The percentage of the sample with different weight classifications as a function of sex and pubertal status is shown in Figure 1.

Table 1 shows the differences between the three weight status groups in body composition and physical fitness, separated by sex and age status (the four subsamples: prepubertal boys, pubertal boys, prepubertal girls, and pubertal girls). Significant differences in all body composition variables were found in the four subsamples. The obese group showed higher body mass, BMI fat mass (kg and %) and trunk fat mass (kg and %), and less lean mass (%) than the overweight group (*p* < 0.001; ES: 0.40 to 2.80), and higher body mass, BMI fat mass (kg and %) and trunk fat mass (kg and %), and less lean mass (kg and %) than the normoweight group (*p* < 0.001; ES: 0.72 to 5.05). Furthermore, the overweight group showed higher body mass, BMI fat mass (kg and %) and trunk fat mass (kg and %), and less lean mass (kg and %) than the normoweight group (*p* < 0.001; ES: 0.72 to 2.90).

The normoweight group performed significantly better in the 20 mSRT (stages-pc) than the overweight group (*p* < 0.05; ES: 0.50 to 0.67) and the obese group (*p* < 0.001; ES: 1.42 to 1.75). Moreover, the overweight group performed better than the obese group in all cases except pubertal girls (*p* < 0.001; ES: 0.82 to 1.03). The overweight group showed significantly higher results than the normoweight group in handgrip (kg-pc) in prepubertal and pubertal boys (*p* < 0.01; ES: 0.31 to 0.53). Similarly, the obese group revealed significantly higher results than the normoweight group in handgrip (kg-pc) in prepubertal boys (*p* < 0.01; ES: 0.37 to 0.59). Instead, the normoweight group showed significantly lower results than the overweight group in handgrip (kg-pc) in prepubertal girls (*p* < 0.01; ES: 0.43 to 0.52). No significant differences in pubertal girls were found (*p* < 0.05). Finally, the normoweight group and the overweight group showed higher results than the obese group in CMJ (pc) in prepubertal boys and pubertal boys (*p* < 0.05; ES: 0.58 to 1.10). On the other hand, the normoweight group and the overweight group presented significantly higher results in both total CMJ (cm) and CMJ (pc) in prepubertal girls (*p* < 0.05; ES: 0.64 to 1.23) and the normoweight group also respect to the overweight group (*p* < 0.05; ES: 0.35 to 0.55). On the contrary, in pubertal girls, significant differences were found only in CMJ (cm) in the normoweight group with respect to the obese group (*p* < 0.001; ES: 0.95).

### 3.2. Differences in Body Composition and Physical Fitness between Adherence to the MD Groups (KIDMED Score)

**Figure 2:**
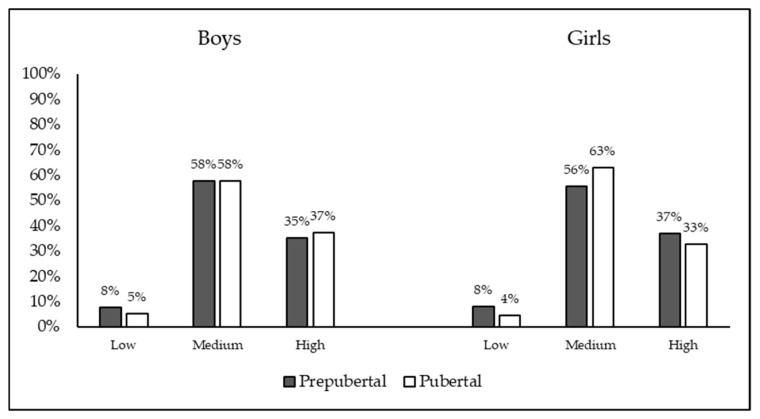
Percentage of the sample with different KIDMED classifications as a function of sex and pubertal status.

According to the results, 6.7% (*n* = 112) of the participants were classified as low adherence to the MD, 57.6% (*n* = 966) were classified as medium adherence to the MD and 35.7% (*n* = 598) were classified as high or optimal adherence to the MD. The percentage of the sample with different weight classifications as a function of sex and pubertal status is shown in Figure 2.

Table 2 shows the differences among the three groups of adherence to the MD (low, medium or high) in body composition and physical fitness variables, separated by sex and age status (the four sub-samples: prepubertal boys, pubertal boys, prepubertal girls and pubertal girls). The high adherence group showed higher body mass than the low adherence group in prepubertal boys (*p* < 0.001; ES: 0.47). Furthermore, the high adherence group reported higher lean mass (kg) than the medium and high groups in prepubertal boys (*p* < 0.05; ES: 0.21 to 0.52). The medium or moderates group also showed significantly higher lean mass (kg) than the low group in prepubertal boys (*p* = 0.33; ES: 0.33). No significant differences in pubertal boys, prepubertal girls and pubertal girls were found in body composition variables (*p* < 0.05). 

Regarding physical fitness parameters, the medium and high adherence groups performed significantly better than the low adherence group in the 20 mSRT (stages) in prepubertal boys (*p* < 0.05; ES: 0.39 to 0.51). Furthermore, the high adherence group performed significantly better than the medium adherence group in pubertal boys in both the 20 mSRT (stages) (*p* = 0.002; ES: 0.35) and the 20 mSRT (pc) (*p* = 0.018; ES: 0.29). No significant differences in prepubertal and pubertal girls were found in both 20 mSRT variables (*p* < 0.05). In handgrip (kg) and handgrip (pc) the high adherence group showed higher values than the medium and low adherence groups in prepubertal boys (*p* < 0.05; ES: 0.21 to 0.56). The medium adherence group also performed significantly greater handgrip (kg) than the high adherence group in prepubertal boys (*p* = 0.34; ES: 0.34). In pubertal boys, the high adherence group showed significantly higher values in handgrip (kg) and handgrip (pc) than the medium adherence group (*p* < 0.01; ES: 0.31 to 0.33). No significant differences in prepubertal and pubertal girls were found in handgrip variables (*p* < 0.05). Finally, prepubertal girls with medium adherence showed higher CMJ (pc) than the high adherence group (*p* = 0.44; ES: 0.31). No significant differences in prepubertal boys, pubertal boys and pubertal girls were found in handgrip variables (*p* < 0.05).

### 3.3. Relationship of Body Composition and Physical Fitness with BMI and KIDMED Score

Table 3 shows the relationship of body composition, physical fitness, and the KIDMED score with BMI and fat mass and the relationship of body composition and physical fitness with the KIDMED score, controlled by sex, pubertal status and age. 

The models estimated for BMI (kg/m^2^) and fat mass explained more than 80% of the variance. The 20 msRT had a negative relationship with BMI and fat mass (*p* < 0.01). However, handgrip and CMJ had a positive relationship with BMI and a negative relationship with fat mass (*p* < 0.05). BMI and fat mass were positively related (*p* < 0.01). The Beta coefficients showed that fat mass and handgrip are the variables with more impact upon BMI regression and BMI and CMJ for fat mass regression.

Contrary to the previous models, the estimation of the KIDMED score explained a very little percentage of variance, with the R^2^ below 0.1. However, the 20 msRT presented a positive and significant relationship with the KIDMED score (*p* < 0.01). The Beta coefficients showed that the 20 mSRT is the more important variable.

## 4. Discussion

The current study attempted to compare body composition and physical fitness according to the weight status (normoweight, overweight and obese) and the level of adherence to the MD (low, medium or high) in 1676 physically active children and adolescents, as a function of sex and pubertal status. Furthermore, the relationships between body composition and physical fitness with the BMI and KIDMED score were also analyzed. This study suggests that weight status is a fundamental factor for health, which is significant in body composition variables as well as in the effect on physical fitness. The results show that normoweight young and adolescent active people showed better values of body composition and physical fitness. Furthermore, fat mass is negatively associated with physical fitness. In the same way, high or optimal adherence to the MD revealed better performance in the different physical fitness parameters, especially in the population of boys. For this reason, it is important to establish specific initiatives and strategies that increase public awareness about this health problem in children and adolescents, leading to changes in nutritional and active-healthy habits.

A total of 26.6% and 16.9% of participants in this study were overweight and obese, respectively. This shows that almost half of the study sample demonstrated values related to obesity, which agrees with other studies [21,47,48]. Even though the participants were physically active, these results may be due to the influential and combination of various factors, such as inadequate daily eating patterns; increased time for sedentary behavior, characterized often as screen-based media-use behaviors [49]; not performing the minimum physical activity recommended by the WHO [50]; or insufficient sleep, which may negatively affect body weight [51]. In relation to body composition variables, significant differences between sex and age groups in body mass, BMI, fat mass, trunk fat mass, and lean mass were found. Our results showed a higher value in the aforementioned variables in the obese group. Similar results have been previously evidenced [21], and several factors, such as changes in dietary patterns, have been shown and justified in the aetiology of obesity [52]. Besides, girls showed a higher percentage of body fat mass compared to boys, which may also be due to a higher level of a sedentary lifestyle and, therefore, the lesser practice of physical activity of the girls, which are determining factors of NCDs [53]. Conversely, the boys showed a higher percentage of lean body mass, which gives them a higher ability to achieve greater levels of cardiorespiratory fitness and strength [54]. Thus, in childhood as well as adolescence it is essential to acquire a balanced diet that may contribute to optimal health, growth and cognitive development. 

When analyzing the results of the differences in weight status regarding the performance in the physical fitness parameters, a higher value in cardiorespiratory fitness and muscular strength was observed in the different groups. The normoweight group showed a higher value in 20 mSRT and CMJ with respect to the overweight and obese groups. Instead, in handgrip strength, the overweight and obese groups had a higher value for both boys and girls. It is known that participants with obesity have greater extra weight and must be able to support it by developing greater muscle mass and, consequently, more strength [55,56]. Based on anthropometric measurements, the highest values in the handgrip test in overweight and obese adolescents could be justified by the different bone and muscle development corresponding to the different growth rates [57].

The results acquired through the KIDMED questionnaire (low, 6.7%; medium, 57.6%; and high, 35.7% adherence to the MD) were similar with other studies carried out in Mediterranean countries [37,58]. Adherence to the MD has been justified with a high level of scientific evidence its effectiveness in preventing of cardiovascular diseases [59]. Our findings showed that 93.3% of the studied population had medium and high adherence to the MD, revealing a clear tendency to maintain or increase patterns related to this type of diet. Thus, a higher score on the KIDMED questionnaire is associated with a more active lifestyle [60]. 

Regarding body composition variables, there were no significant differences between boys and girls in the adherence to the MD, due mainly to the association with physical activity and, possibly, diet adequacy [39]. On the other hand, when analyzing the results of the different tests of physical fitness regarding adherence to the MD, it is confirmed that the athletes with the greatest adherence to the MD are those that presented the highest performance in the different physical fitness tests, especially in boys. The results reveal how high adherence to the MD seems to be related to higher cardiorespiratory fitness, as has been demonstrated in previous studies [61,62]. This fact could be explained by a better body and cardiorespiratory profile caused by the physical activity that the participants practice [59,63], since the higher practice of physical activity (as a result of an active lifestyle) could increase the physical level [64]. Similarly, this occurs with the results of the handgrip test, with a positive association between upper-body muscular strength and a high adherence to the MD. A prior study [65] revealed that this combination seems to provide the highest protection against cardiometabolic risk. Significant differences were not found in the CMJ, except in the girls, where the medium adherence group revealed higher lower-body muscular strength than the group with high or optimal adherence. However, the results show a very unclear relationship between body composition and physical fitness with the KIDMED score, measured at the −4 to 12 score. Therefore, the KIDMED questionnaire should be used mainly to classify participants into the three levels of adherence to the MD (low, medium or high), and not as a continuous scale, in the active population.

The current study presented different limitations. Firstly, since it is a study that presents a cross-sectional design, the contributions cannot be attributed to plausible causes. However, they can be used as indications for future research. Secondly, although the KIDMED score is the instrument most widely used to understand adherence to the MD, it may have been interesting to know different information about the frequency of consumption of certain foods in the Mediterranean pattern—this should be taken into account in future research. In addition, future studies should take into account the socioeconomic status of the families, as this variable could influence the degree of association with the MD. Finally, this study identifies critical behaviors in a sample of physically active Spanish children and adolescents, contributing to the development of public sports policies and public health programs that are needed to combat the adverse health effects of inappropriate nutritional habits.

## 5. Conclusions

The results suggest that the normoweight status showed better parameters of body composition and physical fitness than the overweight and obese status in a young and adolescent active population. Boys classified with high adherence to the MD also showed better values in physical fitness and body composition variables. However, the differences based on the level of adherence to the MD in girls are less significant. Nevertheless, considering the general results of the study, the authors point out the importance of the development of proposals and applications on future strategies of educational and health programs, which should be based on the prevention of overweightness and obesity and improving good nutritional and active-healthy lifestyle habits in younger generations.

## Figures and Tables

**Table 1:** Differences in body composition, physical fitness parameters and KIDMED scores among weight status groups.

	**Boys**																	
	**Prepubertal**									**Pubertal**								
	**Normoweight**			**Overweight**			**Obese**			**Normoweight**			**Overweight**			**Obese**		
Body mass	32.48	±	7.37 ^bc^	41.12	±	9.23 ^c^	50.56	±	12.28	51.71	±	8.66 ^bc^	67.29	±	10.20 ^c^	78.45	±	11.61
BMI (kg/m^2^)	16.66	±	1.42 ^bc^	19.95	±	1.54 ^c^	24.28	±	3.00	19.11	±	1.68 ^bc^	23.69	±	1.47 ^c^	28.72	±	2.12
Fat mass (kg)	6.25	±	1.85 ^bc^	10.20	±	2.91 ^c^	16.86	±	6.47	9.01	±	2.41 ^bc^	15.85	±	3.66 ^c^	25.20	±	5.66
Fat mass (%)	19.16	±	3.12 ^bc^	24.67	±	3.43 ^c^	32.55	±	6.11	17.32	±	3.20 ^bc^	23.55	±	4.10 ^c^	32.21	±	5.70
Lean mass (kg)	24.79	±	5.69 ^bc^	29.24	±	6.63 ^c^	31.96	±	6.84	40.50	±	6.70 ^bc^	48.83	±	7.78	50.55	±	8.84
Lean mass (%)	76.37	±	2.92 ^bc^	71.25	±	3.24 ^c^	63.94	±	5.87	78.40	±	3.03 ^bc^	72.56	±	3.90 ^c^	64.35	±	5.45
Trunk fat (kg)	2.71	±	0.96 ^bc^	4.54	±	1.52 ^c^	7.26	±	2.79	3.98	±	1.30 ^bc^	7.33	±	2.04 ^c^	11.76	±	2.73
Trunk fat (%)	14.37	±	3.49 ^bc^	19.82	±	4.28 ^c^	27.15	±	6.75	14.06	±	3.48 ^bc^	20.62	±	4.82 ^c^	29.52	±	6.34
20 mSRT (stages)	5.86	±	2.16 ^bc^	4.77	±	1.59 ^c^	3.29	±	1.46	8.21	±	1.89 ^bc^	7.21	±	2.13 ^c^	5.24	±	1.97
20 mSRT (pc)	76.81	±	19.76 ^bc^	63.64	±	20.52 ^c^	44.95	±	24.12	66.15	±	20.04 ^bc^	53.75	±	24.40 ^c^	30.82	±	20.33
Handgrip (kg)	17.11	±	5.86 ^bc^	18.98	±	6.32	19.43	±	6.70	31.04	±	8.86 ^b^	35.74	±	9.50	33.91	±	9.18
Handgrip (pc)	47.66	±	28.22 ^bc^	57.17	±	29.07	63.97	±	26.62	44.19	±	28.29 ^b^	59.68	±	29.72	54.53	±	29.23
CMJ (cm)	22.11	±	4.84 ^c^	21.06	±	4.61	17.63	±	4.08	32.29	±	6.10 ^c^	31.60	±	6.18	26.33	±	5.83
CMJ (pc)	57.62	±	25.44 ^bc^	47.81	±	25.28 ^c^	31.91	±	21.27	35.71	±	19.20 ^c^	32.86	±	21.70^c^	21.94	±	16.21
	**Girls**																	
	**Prepubertal**									**Pubertal**								
	**Normoweight**			**Overweight**			**Obese**			**Normoweight**			**Overweight**			**Obese**		
Body mass	31.45	±	8.08 ^bc^	41.07	±	9.74 ^c^	48.66	±	13.20	50.93	±	7.44 ^bc^	61.57	±	7.95 ^c^	72.95	±	7.04
BMI (kg/m^2^)	16.55	±	1.75 ^bc^	20.64	±	1.74 ^c^	24.18	±	2.79	19.95	±	2.03 ^bc^	24.45	±	1.70 ^c^	29.48	±	2.52
Fat mass (kg)	7.22	±	2.36 ^bc^	11.97	±	3.44 ^c^	17.17	±	6.43	13.48	±	3.16 ^bc^	20.23	±	4.62 ^c^	29.16	±	5.90
Fat mass (%)	22.74	±	3.06 ^bc^	28.93	±	3.22 ^c^	34.68	±	4.65	26.21	±	3.54 ^bc^	32.68	±	4.89 ^c^	39.65	±	4.51
Lean mass (kg)	22.95	±	5.71 ^bc^	27.59	±	6.38	29.87	±	7.13	35.53	±	4.62 ^bc^	39.23	±	4.99	41.56	±	2.18
Lean mass (%)	73.20	±	2.87 ^bc^	67.36	±	3.06 ^c^	61.94	±	4.40	70.01	±	3.35 ^bc^	63.89	±	4.64 ^c^	57.28	±	4.27
Trunk fat (kg)	3.03	±	1.07 ^bc^	5.30	±	1.56 ^c^	7.70	±	2.81	5.84	±	1.64 ^bc^	8.76	±	2.20 ^c^	12.41	±	2.22
Trunk fat (%)	16.75	±	4.06 ^bc^	23.05	±	4.14 ^c^	28.74	±	5.05	20.46	±	3.96 ^bc^	26.29	±	5.18 ^c^	33.02	±	4.50
20 mSRT (stages)	4.33	±	1.61 ^bc^	3.48	±	1.33 ^c^	2.46	±	0.97	5.34	±	1.58 ^bc^	4.31	±	1.46	3.27	±	0.98
20 mSRT (pc)	81.12	±	18.36 ^bc^	69.09	±	22.04 ^c^	50.79	±	22.46	67.93	±	24.42 ^bc^	52.63	±	25.95	32.27	±	21.26
Handgrip (kg)	15.27	±	5.33 ^b^	17.57	±	5.48	16.41	±	5.16	24.43	±	5.15	24.60	±	4.01	26.46	±	4.65
Handgrip (pc)	47.54	±	31.04 ^b^	63.00	±	28.17	58.67	±	30.32	46.32	±	27.57	48.12	±	26.36	56.38	±	30.39
CMJ (cm)	20.83	±	4.67 ^bc^	19.31	±	4.04 ^c^	16.36	±	3.63	26.39	±	4.50 ^c^	23.92	±	5.03	20.69	±	7.54
CMJ (pc)	58.87	±	24.62 ^bc^	45.41	±	24.62 ^c^	30.98	±	20.59	43.65	±	22.41	32.94	±	23.92	23.75	±	27.22

Data expressed as mean ± standard deviation. Abbreviations: BMI, body mass index; 20 mSRT, 20-m shuttle run test; Handgrip, handgrip strength; CMJ, countermovement jumps test; pc: percentile. ^b^ Significantly different from overweight group (*p* ≤ 0.05). ^c^ Significantly different from obese group (*p* ≤ 0.05).

**Table 2:** Differences in body composition and physical fitness parameters among different adherences to the Mediterranean diet.

	**Boys**																	
	**Prepubertal**									**Pubertal**								
	**Low**			**Medium**			**High**			**Low**			**Medium**			**High**		
Body mass	34.80	±	12.18 ^c^	38.37	±	11.42	40.35	±	11.63	59.44	±	14.97	57.72	±	13.81	60.61	±	13.14
BMI (kg/m^2^)	18.52	±	3.46	19.19	±	3.47	19.44	±	3.71	21.50	±	3.67	21.30	±	3.87	21.63	±	3.59
Fat mass (kg)	8.48	±	4.66	9.55	±	5.38	10.05	±	5.84	12.62	±	7.51	12.41	±	6.39	13.19	±	6.34
Fat mass (%)	23.11	±	5.99	23.66	±	6.46	23.58	±	7.04	20.32	±	6.53	20.56	±	6.36	20.97	±	6.31
Lean mass (kg)	24.87	±	7.72 ^bc^	27.28	±	6.75 ^c^	28.66	±	6.74	44.44	±	8.94	42.98	±	8.49	45.00	±	8.22
Lean mass (%)	72.57	±	5.58	72.19	±	6.05	72.29	±	6.61	75.61	±	6.23	75.34	±	6.00	74.98	±	5.98
Trunk fat (kg)	3.73	±	2.12	4.18	±	2.37	4.35	±	2.62	5.60	±	3.58	5.65	±	3.16	5.99	±	3.14
Trunk fat (%)	18.40	±	7.13	18.69	±	6.54	18.64	±	7.20	17.00	±	6.87	17.49	±	6.84	17.84	±	6.71
20 mSRT (stages)	4.16	±	2.01 ^bc^	4.96	±	2.03	5.25	±	2.27	7.17	±	1.87	7.33	±	2.09 ^c^	8.09	±	2.30
20 mSRT (pc)	61.29	±	27.53	66.32	±	23.77	67.36	±	24.69	55.95	±	20.16	56.25	±	24.01 ^c^	63.17	±	24.47
Handgrip (kg)	15.65	±	6.46 ^bc^	17.83	±	6.17 ^c^	19.17	±	6.18	32.67	±	7.92	31.41	±	8.93 ^c^	34.33	±	9.72
Handgrip (pc)	47.88	±	31.91 ^c^	52.04	±	28.04 ^c^	58.06	±	29.11	51.29	±	22.33	45.34	±	28.52 ^c^	55.20	±	30.95
CMJ (cm)	19.15	±	3.95	20.78	±	5.08	21.10	±	4.84	32.62	±	6.66	30.61	±	6.39	31.92	±	6.36
CMJ (pc)	53.08	±	24.83	48.45	±	26.62	48.21	±	26.76	40.00	±	21.60	31.36	±	20.04	34.12	±	19.33
	**Girls**																	
	**Prepubertal**									**Pubertal**								
	**Low**			**Medium**			**High**			**Low**			**Medium**			**High**		
Body mass	33.83	±	7.89	35.97	±	10.94	37.91	±	12.45	54.48	±	15.17	55.79	±	10.90	54.84	±	9.08
BMI (kg/m^2^)	17.96	±	2.00	18.54	±	3.20	19.23	±	3.86	21.95	±	5.29	21.96	±	3.99	21.69	±	3.09
Fat mass (kg)	8.52	±	2.44	9.60	±	4.53	10.72	±	5.87	17.32	±	9.36	16.61	±	6.75	16.21	±	5.24
Fat mass (%)	25.08	±	2.82	25.74	±	5.23	26.92	±	6.22	30.13	±	8.27	28.83	±	6.13	28.99	±	5.40
Lean mass (kg)	23.98	±	5.49	25.00	±	6.66	25.77	±	6.94	35.27	±	6.41	37.19	±	4.96	36.65	±	4.88
Lean mass (%)	71.00	±	2.66	70.37	±	4.93	69.25	±	5.86	66.33	±	7.88	67.54	±	5.81	67.36	±	5.10
Trunk fat (kg)	3.69	±	1.09	4.15	±	2.10	4.68	±	2.67	7.68	±	4.62	7.05	±	2.87	7.19	±	2.45
Trunk fat (%)	20.14	±	5.95	19.66	±	5.54	20.96	±	6.84	24.38	±	9.11	22.59	±	6.00	23.49	±	5.42
20 mSRT (stages)	3.81	±	1.61	3.88	±	1.66	3.78	±	1.51	4.75	±	1.44	5.01	±	1.56	4.85	±	1.85
20 mSRT (pc)	73.35	±	21.20	73.96	±	22.59	73.05	±	23.03	58.33	±	26.96	62.89	±	25.25	59.62	±	29.80
Handgrip (kg)	15.43	±	4.91	15.92	±	5.48	16.44	±	5.47	26.12	±	5.48	24.47	±	5.00	24.87	±	4.67
Handgrip (pc)	47.31	±	28.33	54.63	±	31.06	52.77	±	31.21	57.50	±	31.90	45.09	±	27.67	51.40	±	26.72
CMJ (cm)	19.69	±	4.28	20.06	±	4.53	19.20	±	4.81	27.10	±	3.50	24.96	±	5.29	25.66	±	5.47
CMJ (pc)	50.95	±	21.43	54.11	±	25.32 ^c^	45.82	±	27.51	60.00	±	20.00	37.65	±	23.12	40.00	±	25.76

Data expressed as mean ± standard deviation. Abbreviations: Low, low adherence to the MD group; Medium: moderate or medium adherence to the MD group; High, optimal or high adherence to the MD group; BMI: body mass index; 20 mSRT: 20-m shuttle run test; Handgrip: handgrip strength; CMJ: countermovement jumps test, pc: percentile; ^b^ Significantly different from medium adherence to the MD group (*p* ≤ 0.05). ^c^ Significantly different from high adherence to the MD group (*p* ≤ 0.05).

**Table 3:** Regression models for BMI fat mass, and KIDMED score.

	BMI (kg/m^2^)			Fat Mass (%)			KIDMED Score		
	B	RSE	Beta	B	RSE	Beta	B	RSE	Beta
Boys (reference variable)	−			−			−		
Girls	−1.85	(0.12) **	−0.22	3.15	(0.20) **	0.22	0.20	(0.18)	0.04
Prepubertal (reference variable)	−			−			−		
Pubertal	0.46	(0.20) *	0.05	−1.23	(0.36) **	−0.08	−0.80	(0.25) **	−0.16
Age (years)	0.27	(0.04) **	0.19	−0.01	(0.07)	−0.01	0.10	(0.06)	−0.13
BMI (kg/m^2^)	−			1.46	(0.04) **	0.84	0.02	(0.04)	−0.03
Fat mass (%)	0.46	(0.01) **	0.80	−			0.01	(0.02)	−0.04
KIDMED Score	0.01	(0.02)	0.01	0.02	(0.04)	0.01	-		
20 mSRT (stages)	−0.15	(0.04) **	−0.09	−0.22	(0.06) **	−0.08	0.13	(0.05) **	0.14
Handgrip (kg)	0.13	(0.01) **	0.28	−0.11	(0.02) **	−0.15	0.01	(0.02)	0.04
CMJ (cm)	0.04	(0.02) *	0.07	−0.23	(0.02) **	−0.23	−0.02	(0.02)	−0.08
Constant	4.90	(0.49) **	−	1.28	(0.87) **	−	5.28	(0.56) **	−
R^2^	0.84			0.83			0.03		
F	438.02 **			548.74 **			3.22 **		

Abbreviations: B: Non-Standardized Coefficients; RSE: Robust Standard Errors; Beta: Beta-Standardized Coefficients; BMI: body mass index; 20 mSRT: 20-m shuttle run test; Handgrip: handgrip strength; CMJ: countermovement jumps test; pc: percentile. * *p* < 0.05; ** *p* < 0.01.
